# A correlation analysis of HHV infection and its predictive factors in an HIV‐seropositive population in Yunnan, China

**DOI:** 10.1002/jmv.25609

**Published:** 2019-11-18

**Authors:** Li Ren, Binghui Wang, Zhijiang Miao, Pan Liu, Shiyi Zhou, Yun Feng, Shuting Yang, Xueshan Xia, Kunhua Wang

**Affiliations:** ^1^ Department of Obstetrics and Gynecology The First People's Hospital of Yunnan Province Kunming China; ^2^ Faculty of Life Science and Technology Kunming University of Science and Technology Kunming China; ^3^ Department of Gastroenterology and Hepatology Erasmus MC‐University Medical Center Rotterdam The Netherlands; ^4^ Department of Urology Yan'an Hospital of Kunming Chenggong Hospital Kunming China; ^5^ Department of Gastrointestinal Surgery, Kunming Engineering Technology Center of Diagnosis and Treatment of Digestive Diseases, Yunnan Institute of Digestive Disease The First Affiliated Hospital of Kunming Medical University Kunming China

**Keywords:** China, highly active antiretroviral therapy (HAART), human herpesviruses (HHVs), human immunodeficiency virus (HIV)

## Abstract

Human herpesviruses (HHVs) have a particularly high prevalence in certain high‐risk populations and cause increased morbidity and mortality in patients with acquired immunodeficiency syndrome (AIDS). Screening and treating subclinical HHV infections reduce human immunodeficiency virus (HIV) infection incidence, disease progression, and transmission. However, there are few studies on HHVs, HIV coinfection rates, and their related risk factors. We aimed to clarify the prevalence of all eight HHVs in peripheral blood samples collected from HIV‐positive patients, and explore the association of HHV infection in HIV‐positive patients in an HIV‐seropositive population in Yunnan. We recruited 121 HIV‐positive patients with highly active antiretroviral therapy (HAART) and 45 healthy individuals. All the eight HHVs were detected using polymerase chain reaction and their epidemiological information and clinical data were collected and statistically analyzed. A high prevalence of HHVs (89.3%) was observed in individuals with HIV infections and with herpes simplex virus (HSV)‐2 (65.3%), and HSV‐1 (59.5%) being the most common. Coinfection with more than two different HHVs was more common in patients with HIV infections receiving HAART (72.7%) than in healthy controls. Older age, being married, higher HIV‐1 plasma viral loads, and use of antiviral protease inhibitors were independently correlated with an increased frequency of HHVs, but we found no association with CD4 count, WHO HIV clinical stage, and HIV infection duration. Our findings are of great significance for the prevention of HHV opportunistic infection in patients with AIDS and their clinical treatment.

## Highlight

- HHV co‐infection in AIDS patients deserves attention during the course of antiviral therapy. Suppressing HHV recurrences had a clinically significant effect on prolonging survival in some cohorts of HIV‐infected patients and may be an effective intervention in reducing HIV spread in China.

## 1 INTRODUCTION

Human immunodeficiency virus (HIV) mainly infects helper T cells of the immune system and eventually leads to acquired immunodeficiency syndrome (AIDS).^1^ HIV infection is becoming a global health issue due to high morbidity and mortality, and comparatively the situation may be worse in Yunnan. By the end of 2016, approximately 93 437 people were estimated to be living with HIV in Yunnan. This province has been considered the epicenter of HIV‐1 in China, and was the first to conduct highly active antiretroviral therapy (HARRT) in China.^2^ The course of infection of HIV‐1 is characterized by a long interval between initial infection and the onset of serious symptoms as T cell numbers are gradually reduced. HIV infection is associated with an increased risk for human herpesviruses (HHVs) and related diseases, such as AIDS‐associated oral lymphoma and oral hairy leukoplakia.^3^

HHVs belong to the *Herpesviridae* family and are widely distributed viruses that cause benign and malignant disease in animals and humans. They are ubiquitous and have a particularly high prevalence in certain high‐risk populations, causing increased morbidity and mortality in patients with AIDS.^4^ There are eight known herpes family viruses associated with human infection: herpes simplex virus (HSV) types 1 and 2, varicella‐zoster virus (VZV), Epstein‐Barr virus (EBV), cytomegalovirus (CMV), human herpesvirus 6 (HHV‐6), human herpesvirus 7 (HHV‐7), and human herpesvirus 8 (HHV‐8). These viruses have both epidemiologic and clinical interactions with HIV‐1, which are associated with serious opportunistic infections and their reactivation predicts poor outcome.^5^, ^6^

Normal host immune response may control and limit HHV replication, but reactivation of latent infection can result in severe opportunistic infections with high morbidity and mortality in immunocompromised subjects.^7^, ^8^ Furthermore, some HHVs might interact with HIV‐1 or have a role as a cofactor for HIV‐1 progression, accelerating the replication of HIV‐1.^9^ At the same time, HIV‐1 can facilitate acquisition and reactivation of HHVs as HIV‐1 and HHVs are involved in a vicious cycle.^10^ Prevalence rates and viral loads are influenced by the biological properties of the virus, method of detection, frequency of sampling, whether symptomatic or not, social behaviors, and immunological status of the patient.^11^ Detection using polymerase chain reaction (PCR) assay shows that the frequencies of these viruses are wide‐ranging across a variety of clinical samples^12^, ^13^, ^14^ and are influenced by the degree of immunodepression,^15^, ^16^ geography, and behavioral risk factors.^17^, ^18^, ^19^ Suppression of HHV recurrence by cotherapy had a clinically significant effect on prolonged survival in some cohorts of HIV‐infected patients.^20^

Zhaotong is located on a drug transmission route far from the border between China and other Southeast Asian countries. It may be the smallest affected area of the Yunnan Province to have an external strain HIV‐1 that is indigenous. The seroprevalence rates and predictive factors of HHV in the setting of HIV‐1 infections in Zhaotong have not been explored. In this study, we aimed to identify the prevalence of all eight HHVs in peripheral blood samples collected from HIV‐positive patients and explore the association of HHV infection in HIV‐positive patients while adjusting for potential confounding factors.

## 2 METHODS

### 2.1 Study participants

A total of 121 HIV‐positive patients were randomly selected from 2517 cases of HIV infection that were treated in The First People's Hospital of Zhaotong City from May 2015 to February 2016. At the same time, we recruited a control group of 45 healthy, HIV‐negative subjects, matched for age at the time of examination. The inclusion criterion for both groups was that they be without symptoms of acute illness (fever, headache, sore throat, body aches, and diarrhea) and malignancy at the time of enrollment. The exclusion criterion included pregnancy, use of immunosuppressant medications, and administration of antiherpetic therapy within 1 week before the date of study enrollment. Plasma samples from all subjects were stored at − 80°C until DNA was extracted. The study was approved by the Ethics Committee of The First Affiliated Hospital of Kunming Medical University. Verbal consent was obtained from all the participants before enrollment in the study.

### 2.2 HHV infection detection

Viral DNA was extracted from 200 μL of plasma using the TIANamp Genomic DNA kit (TIANGEN, Beijing, China) according to the manufacturer's instructions. The DNA was subjected to nested PCR to amplify the fragments of HSV‐1, HSV‐2, VZV, EBV, HCMV, HHV‐6, HHV‐7, and HHV‐8 with the outer and inner oligonucleotide primers designed for this study. Each sample was tested in duplicate and scored as positive or negative in case of concordant results. When results were discordant, two new aliquots were examined, and the sample was regarded as positive if at least two of four examinations were positive. PCR products were run on 2% agarose gel stained with ethidium bromide and visualized under a UV light transilluminator and sent to a commercial sequencing location (Invitrogen, Beijing, China).

### 2.3 Epidemiological information and clinical data

Data on the social‐demographic characteristics of these participants as well as the informed consent to participate in this study were obtained from the enrolled participants during the face‐to‐face interview by the outpatient doctor. Clinical data were also collected from the hospital laboratory information system, including a blood biochemical analytical index and CD4 lymphocyte count.

### 2.4 Statistical analysis

Statistical analyses were conducted using the SPSS software (IBM SPSS Statistics for Windows, Version 19.0; IBM Corp, Armonk, NY). The *χ* ^2^ and Fisher's exact tests were applied to compare the demographic characteristics distribution and seroprevalence rates between the two groups. Logistic regression was used to calculate adjusted odds ratios and 95% confidence intervals on the association of the eight HHVs and multiple HHV infections with socio‐demographic and biological risk factors. The results were considered statistically significant if *P* < .05.

## 3 RESULTS

### 3.1 Demographic characteristics of study participants

The 166 participants were from 11 different areas of the Zhaotong prefecture. Among the HIV‐positive and healthy HIV‐negative groups, the ratio of male to female participants was 77:44 in the HIV‐positive group and 20:25 in the control group. The mean age of the participants was 42 ± 14.0 years in the HIV group and 40 ± 12.2 years in the control group. The majority of the subjects were in the age range 31 to 40 with some over 41 years of age, with a distribution of 38% and 46.3% in the HIV group, and 37.8% and 26.7% in the control group. The majority of the subjects were married, accounting for 73.6% in the HIV group and 66.7% in the control group, followed by unmarried (12.4% vs 28.9%), divorced (7.4% vs 2.2%), widowed (5.8% vs 2.2%), and other/unknown (0.8% vs 0.0%). No significant differences among demographic characteristics were observed between either groups. Sexual transmission was the main HIV infection route and several were infected by sharing drug needles.

### 3.2 HHVs infection status

**Figure 1:**
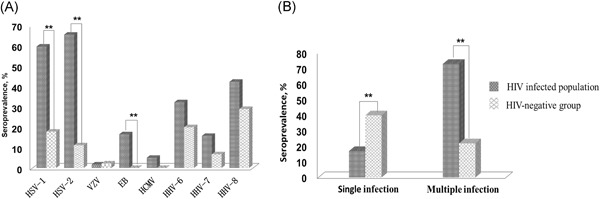
Seroprevalence rates of (A) single or (B) multiple HHV infection in peripheral blood as determined by nested PCR. *χ* ^2^ and Fisher's exact tests were used to test the differences between the HIV group and the control; the significant differences are indicated with asterisks. HHV, human herpesviruse; HIV, human immunodeficiency virus; PCR, polymerase chain reaction

In total, among 108 HIV‐positive individuals were positive for HHV with at least one HHV infection. The prevalence of HHV was higher (89.3%) in peripheral blood of HIV‐positive group than the control group (62.2%). The most prevalent viruses in the HIV group were HSV‐2 (65.3%) and HSV‐1 (59.5%), followed by HHV‐8 (42.2%), HHV‐6 (32.2%), EBV (16.5%), HHV‐7 (15.6%), HCMV (5.0%), and VZV (1.7%). In contrast, HHV‐8 (28.9%) and HHV‐6 (20.0%) were the most prevalent viruses in the control group, followed by HSV‐1 (17.8%), HSV‐2 (11.1%), HHV‐7 (6.7%), and VZV (2.2%), but EBV and HCMV were not detected in this group (Figure 1A). There was not enough data on the seroprevalence of CMV and HCMV for accurate statistical analysis. Statistical analysis indicated that there were significant differences in the presence of HSV‐1 (*P* < .001), HSV‐2 (*P* < .001), and EBV (*P* < .01) between the two groups.

The number of double and triple HHV infections were 33 and 30 in HIV‐positive group, respectively, higher than a single infection. Three cases were found to have six HHV infections (one was negative for VZV and EBV and two showed failure to detect VZV and HCMV). Coinfection with multiple HHVs was more likely to occur in the HIV group than in the control group, especially coinfection with more than two different HHVs (*P* < .001), and this difference was found to be statistically significant. However, the control group was more likely to be coinfected with only one HHV (*P* < .01) (Figure 1B).

### 3.3 Related predictive factors in HIV group

Statistical analysis of demographic and clinical information was performed to further uncover the relationship between different predictive factors and eight HHV infections in HIV‐1‐positive patients.

Age was the most relevant factor for single HHV infections in the HIV group and significant differences were observed for the risk of infection by HHV‐1 (*P* < .001), HSV‐2 (*P* = .049), HHV‐6 (*P* < .01), and HHV‐8 (*P* = .001) at different age stages (Table 1). The infection rates among those older than 31 years were especially higher than among others. Marital status was another leading risk for HHV; infections (HSV‐2, HHV‐6, and HHV‐8) were much higher among married patients. Infections by EBV and HHV‐7 were also associated with different immune statuses. Low CD4 counts were correlated with infection by EBV (*P* = .010) and HHV‐7 (*P* = .044). A noteworthy observation is that HIV viral load only influenced EBV infection, and EBV infection decreased with the increase of HIV viral load (*P* = .013). Current HAART regimens using double nucleoside reverse‐transcriptase inhibitors (NRTIs) and single nonnucleoside reverse‐transcriptase inhibitors (NNRTIs) therapy can slightly increase the risk for infection by HSV‐1 (*P* = .049) and HHV‐8 (*P* = .044). Furthermore, for infection by HSV‐1, males were more at risk than females (*P* = .029), and for infection by EBV, people with HIV‐1 transmitted by intravenous drug use and sexual activity were more likely to be infected (*P* < .001). Surprisingly, there were no significant differences in the seroprevalence of the eight HHVs with different WHO stages and durations of HIV infection, which were thought to be related to organism immunization.

**Table 1:** Single HHVs infection and related predictive factors in HIV group

Characteristics	Seroprevalence rate							
	HSV‐1	HSV‐2	VZV	EBV	HCMV	HHV‐6	HHV‐7	HHV‐8
Total, no. (%)	72 (59.5)	79 (65.3)	2 (1.7)	20 (16.5)	6 (5)	39 (32.2)	19 (15.7)	51 (42.1)
Sex, no. (%)	***P = .029***	*P* = .377	*P* = .533	*P* > .999	*P* = .085	*P* > .999	*P* = .409	*P* = .986
Male	52 (43)	53 (43.8)	2 (1.7)	13 (10.7)	6 (5.0)	25 (20.7)	10 (8.3)	33 (27.3)
Female	20 (16.5)	26 (21.5)	0 (0.0)	7 (5.8)	0 (0.0)	14 (11.6)	9 (7.4)	18 (14.9)
Age, no. (%), y	***P*** **<** ***.001***	***P = .049***	*P* = .645	*P* = .275	*P* > .999	***P < .01***	*P* = .600	***P = .001***
≤20	0 (0.0)	1 (0.8)	0 (0.0)	2 (1.7)	0 (0.0)	0 (0.0)	1 (0.8)	0 (0.0)
21‐30	4 (3.3)	8 (6.6)	0 (0.0)	2 (1.7)	0 (0.0)	2 (1.7)	1 (0.8)	1 (0.8)
31‐40	26 (21.5)	28 (23.1)	0 (0.0)	5 (4.1)	3 (2.5)	10 (8.3)	6 (5.0)	18 (14.9)
≥41	42 (34.7)	42 (34.7)	2 (1.7)	11 (9.1)	3 (2.5)	27 (22.3)	11 (9.1)	32 (26.4)
Marital status, no. (%)^a^	*P* = .279	***P = .016***	*P* > .999	*P* = .199	*P* > .999	***P = .032***	*P* = .655	***P = .026***
Unmarried	6 (5.0)	5 (4.1)	0 (0.0)	5 (4.1)	0(0.0)	1 (0.8)	1 (0.8)	4 (3.3)
Married	56 (46.3)	61 (50.4)	2 (1.7)	13 (10.7)	5(4.1)	30 (24.8)	16 (13.2)	35 (28.9)
Others/unknown	10 (8.3)	13 (10.7)	0 (0.0)	2 (1.7)	1(0.8)	8 (6.6)	2 (1.7)	12 (9.9)
CD4 (cells/µL), no. (%)	*P* = .089	*P* = .660	*P* > .999	***P = .010***	*P* = .576	*P* = .275	***P = .044***	*P* = .273
≤200	26 (21.5)	25 (20.7)	1 (0.8)	6 (5.0)	2 (1.7)	9 (7.4)	11 (9.1)	14 (11.6)
201‐399	28 (23.1)	31 (25.6)	1 (0.8)	7 (5.8)	4 (3.3)	17 (14.0)	5 (4.1)	26 (21.5)
≥400	16 (13.2)	19 (15.7)	0 (0.0)	2 (1.7)	0 (0.0)	12 (9.9)	3 (2.5)	8 (6.6)
Others/unknown	2 (1.7)	4 (3.3)	0 (0.0)	5 (4.1)	0 (0.0)	1 (0.8)	0 (0.0)	3 (2.5)
WHO stage, no. (%)	*P* = .499	*P* = .226	*P* > .999	*P* = .581	*P* = .663	*P* = .201	*P* = .739	*P* = .981
Stage I/II	51 (42.1)	57 (47.1)	2(1.7)	12 (9.9)	5 (4.1)	30 (24.8)	14 (11.6)	34 (28.1)
Stage III/IV	21 (17.4)	22 (18.2)	0(0.0)	8 (6.6)	1 (0.8)	9 (7.4)	5 (4.1)	17 (14.0)
HIV viral load (copies/mL), no. (%)	*P* = .072	*P* = .051	*P* = .521	***P = .013***	*P* = .340	*P* = .154	*P* = .747	*P* = .914
Undetectable	19 (15.7)	20 (16.5)	0 (0.0)	5 (4.1)	1 (0.8)	14 (11.6)	7 (5.8)	18 (14.9)
<1000	13 (10.7)	13 (10.7)	0 (0.0)	4 (3.3)	0 (0.0)	4 (3.3)	3 (2.5)	8 (6.6)
1000‐9999	19 (15.7)	22 (18.2)	1 (0.8)	3 (2.5)	2 (1.7)	7 (5.8)	5 (4.1)	12 (9.9)
≥10 000	18 (14.9)	19 (15.7)	1 (0.8)	2 (1.7)	3 (2.5)	12 (9.9)	4 (3.3)	9 (7.4)
Others/unknown	3 (2.5)	5 (4.1)	0 (0.0)	6 (5.0)	0 (0.0)	2 (1.7)	0 (0.0)	4(3.3)
Transmission category, no. (%)	*P* = .256	*P* = .863	*P* = .461	***P < .001***	*P* = .816	*P* = .068	*P* = .387	*P* = .912
IDU	8 (6.6)	10 (8.3)	1 (0.8)	8 (6.6)	0 (0.0)	2 (1.7)	4 (3.3)	8 (6.6)
Sexual	57 (47.1)	59 (48.8)	1 (0.8)	8 (6.6)	5 (4.1)	34 (28.1)	12 (9.9)	37 (30.6)
MCT/others/unknown	7 (5.8)	10 (8.3)	0 (0.0)	4 (3.3)	1 (0.8)	3 (2.5)	3 (2.5)	6 (5.0)
HIV infection duration, no. (%), y	*P* = .276	*P* = .342	*P* = .305	*P* = .152	*P* > .999	*P* = .264	*P* = .991	*P* = .226
1	7 (5.8)	8 (6.6)	1 (0.8)	4 (3.3)	1 (0.8)	5 (4.1)	3 (2.5)	6 (5.0)
2	31 (25.6)	31 (25.6)	0 (0.0)	3 (2.5)	2 (1.7)	19 (15.7)	7 (5.8)	24 (19.8)
3	16 (13.2)	18 (14.9)	0 (0.0)	6 (5.0)	1 (0.8)	9 (7.4)	5 (4.1)	12 (9.9)
≥4	17 (14.0)	21 (17.4)	1 (0.8)	6 (5.0)	2 (1.7)	6 (5.0)	4 (3.3)	8 (6.6)
Others/unknown	1 (0.8)	1 (0.8)	0 (0.0)	1 (0.8)	0 (0.0)	0 (0.0)	0 (0.0)	1 (0.8)
Current HAART regimen	***P*** = ***.049***	*P* = .256	*P* > .999	*P* = .431	*P* = .628	*P* = .148	*P* > .999	***P = .044***
Double‐NRTIs + NNRTIs	64 (52.9)	68 (56.2)	2 (1.7)	18 (14.9)	5 (4.1)	32 (26.4)	17 (14.0)	48 (39.7)
Double‐NRTIs + PIs	8 (6.6)	10 (8.3)	0 (0.0)	1 (0.8)	1 (0.8)	7 (5.8)	2 (1.7)	3 (2.5)
Others/unknown	0 (0.0)	1 (0.8)	0 (0.0)	1 (0.8)	0 (0.0)	0 (0.0)	0 (0.0)	0 (0.0)

*Note*: The statistically significant are presented with italic bold.

Abbreviations: HAART, highly active antiretroviral therapy; HIV, human immunodeficiency virus; IDU, intravenous drug use; MCT, mother‐to‐child transmission; NRTIs, nucleoside reverse‐transcriptase inhibitors; NNRTIs, nonnucleoside reverse‐transcriptase inhibitors; PIs, protease inhibitors.

^a^ Others/unknown include divorced, widowed, and others/unknown.

The results of HHV detection showed that patients with HIV were more likely to have greater than 2 HHV infections with different viruses. Multiple HHV infections were found to be significantly associated with age, marital status, CD4 cell counts, and current HAART regimen in HIV‐positive patients, but not related to HIV viral load and WHO stage (Table 2). The proportion of coinfection with multiple viruses was higher in married and older adults (above 41 years old), indicating that they were at a greater risk of having multiple infections. Similarly, different medication regimens of HARRT were also shown to influence multiple HHV infections (*P* = .011). The patients simultaneously using NRTIs with NNRTIs were more likely to have multiple infections. Multiple HHV infections were much higher in patients with lower CD4 cell counts (<400 cells/μL), and especially in subjects with CD4 cell counts of 201 to 399 cells/μL.

**Table 2:** Multiple HHVs infection and related predictive factors in HIV group

Characteristics	Subjects	0	1	≥2	*χ* ^*2*^	*P*
Total, no. (%)	121 (100.0)	13 (10.7)	20 (16.5)	88 (72.7)		
Sex, no. (%)					3.210	.187
Male	77 (63.6)	6 (5.0)	11 (9.1)	69 (49.6)		
Female	44 (36.4)	7 (5.8)	9 (7.4)	28 (23.1)		
Age, no. (%), y					28.960	***<.001***
≤20	5 (4.1)	2 (1.7)	2 (1.7)	1 (0.8)		
21‐30	14 (11.6)	4 (3.3)	4 (3.3)	6 (5.0)		
31‐40	46 (38.0)	7 (5.8)	9 (7.4)	30 (24.8)		
≥41	56 (46.3)	0 (0.0)	5 (4.1)	51 (42.1)		
Marital status, no. (%)^a^					10.333	***.020***
Unmarried	15 (12.4)	4 (3.3)	5 (4.1)	6 (5.0)		
Married	89 (73.6)	9 (7.4)	13 (10.7)	67 (55.4)		
Others/unknown	17 (14.0)	0 (0.0)	2 (1.7)	15 (12.4)		
CD4 (cells/µL), no. (%)					14.154	***.017***
≤200	36 (29.8)	0 (0.0)	9 (7.4)	27 (22.3)		
201‐399	50 (41.3)	9 (7.4)	4 (3.3)	37 (30.6)		
≥400	27 (22.3)	4 (3.3)	4 (3.3)	19 (15.7)		
Others/unknown	8 (6.6)	0 (0.0)	3 (2.5)	5 (4.1)		
WHO stage, no. (%)					1.368	.536
Stage I/II	82 (67.8)	7 (5.8)	14 (11.6)	61 (50.4)		
Stage III/IV	39 (32.2)	6 (5.0)	6 (5.0)	27 (22.3)		
HIV viral load (copies/mL), no. (%)					13.068	.077
Undetectable	38 (31.4)	6 (5.0)	7 (5.8)	25 (20.7)		
<1000	22 (18.2)	5 (4.1)	2 (1.7)	15 (12.4)		
1000‐9999	27 (22.3)	0 (0.0)	5 (4.1)	22 (18.2)		
≥10 000	24 (19.8)	2 (1.7)	2 (1.7)	20 (16.5)		
Others/unknown	10 (8.3)	0 (0.0)	4 (3.3)	6 (5.0)		
Transmission category, no. (%)					2.289	.704
IDU	17 (14.0)	1 (0.8)	3 (2.5)	13 (10.7)		
Sexual	89 (73.6)	10 (8.3)	13 (10.7)	66 (54.5)		
MCT/others/unknown	15 (12.4)	2 (1.7)	4 (3.3)	9 (7.4)		
HIV infection duration, no. (%), y					9.487	.251
1	16 (13.2)	1 (0.8)	4 (3.3)	11 (9.1)		
2	44 (36.4)	3 (2.5)	5 (4.1)	36 (29.8)		
3	29 (24.0)	6 (5.0)	4 (3.3)	19 (15.7)		
≥4	29 (24.0)	3 (2.5)	5 (4.1)	21 (17.4)		
Others/unknown	3 (2.5)	0 (0.0)	2 (1.7)	1 (0.8)		
Current HAART regimen					11.309	***.011***
Double‐NRTIs + NNRTIs	103 (85.1)	9 (7.4)	16 (13.2)	78 (64.5)		
Double‐NRTIs + PIs	14 (11.6)	2 (1.7)	2 (1.7)	10 (8.3)		
Others/unknown	4 (3.3)	2 (1.7)	2 (1.7)	0 (0.0)		

*Note*: The statistically significant are presented with italic bold.

Abbreviations: HAART, highly active antiretroviral therapy; HIV, human immunodeficiency virus; IDU, intravenous drug use; MCT, mother‐to‐child transmission; NRTIs, nucleoside reverse‐transcriptase inhibitors; NNRTIs, nonnucleoside reverse‐transcriptase inhibitors; PIs, protease inhibitors.

^a^ Others/unknown include divorced, widowed, and others/unknown.

## 4 DISCUSSION

Yunnan has been the hardest hit region by HIV in China with more than 90 000 patients with AIDS by 2016, and the first place to introduce HAART in China. Although HARRT has a beneficial treatment effect, opportunistic infections by other viruses, and bacteria present significant difficulties. HHVs have been infecting humans for hundreds of millions of years and can significantly affect morbidity and mortality rates when coinfected with HIV. Screening and treating subclinical HHV infections may offer a slowing of HIV infection, disease progression, and its transmission. This study was initiated to evaluate the seroprevalence rates and correlates of peripheral blood among eight HHVs as well as multiple HHVs in asymptomatic, HIV‐seropositive persons treated with HAART.

The prevalence of HHVs was higher in the peripheral blood of HIV‐positive individuals than in that of the control group. This finding is in accordance with those of other studies, likely because of the high sensitivity of our PCR assay.^21^ The eight HHVs except VZV were more prevalent in HIV‐positive patients than in the control group. Low EBV infection rates were observed both in the general population and patients with HIV, may due to too small amount of serum for extracting nucleic acid. HCMV and VZV were not detected in the control group. The factors consistently and independently associated with HHV infections were male sex, older age, being married, high HIV viral load, mother‐to‐child transmission of HIV, and current use of HAART regimen using PIs instead of NNRTIs.^22^ The relationship between different HHV infections and immune status, WHO HIV clinical stage, and HIV infection duration were not shown in multivariate analysis, in spite of CD4 counts that were significantly associated with the detection of EBV and HHV‐7 in univariate analysis. However, older age was shown to have an important role in the spread of multiple HHV infections in HIV‐positive patients after adjusting for potential confounding factors.

The majority of the HIV‐seropositive groups (72.7%) were positive with at least two detectable HHVs in peripheral blood samples, compared with controls. The higher prevalence in the HIV‐positive group may reflect an altered health status or differences in the HHV seropositivity between groups. Risk factors for VZV and HCMV were not readily evident due to the small number of participants in this study. The prevalence of HSV‐1, HHV‐6, and HHV‐8 infection was higher with increase in age. Moreover, male sex and being married were strongly associated with HSV‐1 infection. These phenomena were probably due to the younger average age at marriage, lower socioeconomic status, crowded living conditions, poor hygiene,^23^ and high levels of concomitant HIV infection in male partners, which may enhance HHVs infectivity of those partners.^24^ Plasma HIV viral load, CD4 count, and HAART therapy influenced each case of herpesvirus infection in different ways.^5^, ^25^ No significant correlations were observed between the rate of HHV shedding and CD4 cell count or plasma HIV‐1 RNA level among the HAART‐treated patients of previous studies^26^, ^27^ and our study.

The consequences of universal access to HAART include a reduction in the number of deaths by AIDS and a reduction of the incidence of opportunistic diseases.^28^ However, in this study, HHV incidence appeared to persist at a higher frequency. Regarding the impact of current HAART regimens for HHV infections, we can conclude that patients treated with PIs instead of NNRTIs were at the highest risk of HHV‐6 infection. As a second‐line treatment, PIs instead of NNRTIs have been included in the HAART regimen for patients with side effects or drug resistance.^29^ These findings suggest that no single type of therapy is likely to reduce reactivation rates among all classes of herpesviruses.^25^ Long‐term follow‐up studies are needed to further confirm the influence on HHV infections by different drugs. An important limitation of this study is that all subjects lack follow‐up serostatus. In the future, longitudinal studies should be performed involving HIV‐positive patients, before and after initiating HAART with sufficient follow‐up and deaths for analysis, to achieve a better understanding of the immune status and disease progression. That might be important for predicting opportunistic infections caused by these viruses. The correlation between the infection rates of eight HHVs genotype and the genotypes of HIV unique epidemic strains in Zhongtong was not clarified.

In conclusion, our study suggests that the frequency of the eight HHVs and occurrence of more than two different HHVs in HIV‐positive peripheral blood with HAART are significantly higher compared with healthy controls when measured in a randomized controlled design. The observations reported herein suggest that the presence of HHVs in peripheral blood is a frequent event in HIV‐infected individuals and increases the risk of multiple infections in these patients, even in the presence of HAART therapy. These data have confirmed that older age, being married, higher HIV‐1 plasma viral load, and use of antiviral medicine PIs were correlated independently with increased frequency of HHVs, but CD4 count, WHO HIV clinical stage, and HIV infection duration were not associated with HHVs. Our findings indicate that suppressing HHV recurrences as a cotherapy to HAART had a clinically significant effect on prolonging survival in some cohorts of HIV‐infected patients and may be an effective intervention in reducing HIV spread in China. Strategies to control HHVs deserve urgent attention and should become part of the HIV‐1 prevention and care package.

## CONFLICT OF INTERESTS

The authors declare that there are no conflict of interests.
